# Monthly Review of Medical and General Science in France and Europe

**Published:** 1869-05-15

**Authors:** 


### CHICAGO MEDICAL JOURNAL.

Vol. XXVI[illegible]—MAY 15, 1869.—No. 10.

### REVIEWS.

### *Monthly Review of Medical and General Science in France and Europe.*

Before entering on our subject we think it advisable to make a few preliminary observations with respect to the programme which we intend to follow.

As your journal is intended especially for the members of the medical profession, we shall devote our principal attention to medical, surgical and pharmaceutical subjects. As the medical body is the only recognized authority on general scientific subjects among the non-professional public, and as it is to physicians that people look for accurate information with regard to facts announced in the non-scientific press, we shall notice all the events in connection with general science which we think worthy of record. We shall abstain as much as possible from criticism, and try to submit facts to the judgment of our readers in such a manner as to enable them to draw their own conclusions.

This much said we commence our review.

An important paper has just been read before the Imperial Academy of Medicine, by Dr. Dupré, Professor of Clinical Surgery at the college at Montpelier. Dr. Dupré divides pleurisitic effusions into three principal orders:

1st. Essentially inflammatory flowings, namely, those which accompany true pleurisies or which succeed them.

2d. Serous accumulations, actual dropsies which occur in the pleura in connection with organic lesions or general deteriorations.

3d. Rheumatismal or sero-plastic effusions. These last are the principal subjects of the paper.

Inflammatory flowings, according to Dr. Dupré, should not be combated by thoracic puncture. In the majority of cases, it might almost be said always to yield to other treatment. Serous accumulations in the pleura can be removed by no other means than puncture. Every thing tends to prove this, as well as the nature of the extravasated serum and its incessant tendency to accumulate, to the great danger of the lesions which have given rise to the extravasion. Rheumatismal or sero-plastic effusions require puncture of the pleura and the evacuations of this extravasated fluid, for these operations are calculated to obviate serious accident, and add nothing to the danger of the situation. Under the name of rheumatismal and sero-plastic effusions, which have been so well described by Mr. Pidaux under the name of latent pleurisy, and which have been so frequently observed by Michel Levy, Tholasan, Lancerotte and Toussagrioes.

The most important accompaniments of these effusions are the following: The beginning is generally accompanied by a slight trembling and a painful feeling of constraint in a part of the thorax. Sometimes, immediately afterwards pains in the joints, or sciatic neuralgia ensue. In other cases the pain in the thorax is very acute, and seriously impedes respiration, but it is superficial and not confined to any particular region, and posseses all the symptoms of pleurodynia, and of rheumatism of the pectoral muscles. It often happens that in the course of this painful state, and frequently when its intensity is decreasing, an overflow of fluid which may or may not become considerable takes place in one of the pleural cavities. This effusion is unaccompanied by cough, oppression or pain. There is no fever, the appetite is unimpaired and sleep uninterrupted. However, a livid paleness, an abnormal motion of the face and neck, sudden interruptions of the breathing in the middle of deep inspirations, and irregular pulse, indicate the existence of the affection. In such cases medical treatment is uncertain in its results, tardy in its effects, and often allows the formation of dangerous lesions. Sudden death not unfrequently occurs. Dr. Dupré advocates puncture in these cases as a certain means of preventing lesions, without adding in any way to the gravity of the disease. After giving the statistics of the operation of thoracic puncture, which we append, Dr. Dupré resumes his conclusions as follows:

1st. There exist idiopathic pleuritic effusions, of which apyrexia, latency and progression are the ordinary characteristics.

2d. They are distinguished from inflammatory effusions, and from hydropic accumulations by the clinical characteristics which make them approach rheumatisms.

3d. The presence of elastic serosity in the pleura, and its prolonged stay there, is a real and considerable danger. It should be removed as promptly as possible, either indirectly by the aid of the medicine, or directly by surgical means.

4th. Thoracic puncture, practiced according to the rules prescribed, is absolutely harmless, and its immediate action and direct effects expose the patient to no danger.

5th. It should be practiced immediately in case of effusions of more than a fortnight's standing, especially those which are situated on the left side, and occupy all the pleural cavity.

6th. In cases where effusions form under the care of a physician, recourse should not be had to this operation before the tenth day, and when they occupy at least two-thirds of the pleural cavity.

These principles put into practice by Dr. Dupré have produced the following results:

| | | |
|---|---|---|
| Patients operated on in the Second Week, 47......... | Cures, | 46. |
| | Deaths, | 1. |
| | | 47. |

Where it was impossible to perform the operation at an early period of the disease, the results were not so favorable.

| | | |
|---|---|---|
| Patients operated in the First Month, 19............... | Cures, | 15 |
| | Deaths, | 4 |
| | | 19 |
| " " " 2d " 8............... | Cures, | 5 |
| | Deaths, | 3 |
| | | 8 |
| " " " 5th " 1............... | Cures, | 1 |
| " " " 17th " 1............... | Cures, | 1 |

This table proves that sero-plastic effusions of very old standing can be cured, and that prompt thoracic puncture is an excellent habitual mode of treatment. The aid of medicine should not, however, be despised. On the contrary, we must rely on it to prepare and assure the success of the operation.

The frequent accidents resulting from the inhalation of chloroform have prompted from their very origin the study of several experimental chemists. Jobert de Lamballe, Robert, Abeille and Siégeoris have written on the happy results of electricity applied as a means of restoring persons asphyxiated by chloroform. With the same object Messrs. Legros and Onimus have experimented on the employment of continuous currents of electricity.

The works of these two last authors have been submitted to the Imperial Society of Surgery. We think that a résumé of the experiments made before a committee of this society, and of the conclusions to which the gentlemen chosen by it to report on this subject have appended the authority of their reputations, will be read with interest.

Three white rats were placed under a bell glass thirty centimetres in height and fifteen in breadth, with a sponge on which a quantity of chloroform had been poured.

The first rat was removed after respiration had entirely ceased, when every trace of sensibility and voluntary movement had disappeared, but before the pulsations of the heart had discontinued. The poles of a voltaic pile were then introduced, one, the negative, into the rectum; the other, the positive into the mouth. The current was maintained during some seconds, after which the wires were withdrawn. In a little while irregular respiratory movements could be perceived, which gradually became more frequent; the pulsations of the heart increased in strength, and gradually sensibility and general movement returned. The animal was completely restored to life.

The second rat was taken from under the bell-glass almost in the same conditions, the movements of the heart being, however, less perceptible. The current failed to restore the animal to life.

The third rat, when taken from under the glass, had ceased to breathe; sensibility and general movements were extinguished; the pulsations of the heart were still perceptible. The application of the current succeeded in reviving it. It is important to observe that all these experiments are applicable to cases of asphyxia by chloroform, and not syncope.

As a means of combating chloroformic asphyxia, galvanization, as proposed by Messrs. Legros and Onimus, appears to the committee to be decidedly efficacious. The animals on which the preceding experiments were made may be considered as devoted to certain death, if left to themselves.

The observations made by Mr. Maurice Perrin on dogs, cats and rabbits prove that animals asphyxiated by chloroform, in these conditions, succumb, if abandoned to the efforts of nature.

The preceding experiments are, we believe, of very serious importance, and calculated to render valuable services to surgical practice. One very important item should be remarked, that the continued current recommended by Messrs. Legros and Onimus only restores the action of the heart when it is made to pass through the cerebro-spinal axis.

The committee next proceeded to experiment upon inductive currents, and obtained as satisfactory results as with the voltaic pile. This fact induced the committee to give the preference to the last; firstly, because an inductive apparatus can be easily procured and is portable; secondly, because induced currents applied to the human heart, diaphragm, and phrenic nerves have received the sanction of experience, while continuous currents, applied to the same organs in the similar cases have not succeeded in producing the same results. Indeed, Friedberg, of Berlin, and Wide, of Hargrave, have successfully employed faradization of the phrenic nerves. Maurice, of the Royal Berg Hospital, has saved a man by passing a current through the elongated ganglion in communication with the epigsatrium. Luimssen and Duchesse de Boulogne restored to life a man who had asphyxiated himself with the vapor of charcoal, by means of faradisation of the epigastrium. Whatever may be thought of previous experiments and of the probable advantages of inductive electricity, Messrs. Legros and Onimus must be allowed the credit of having placed at the disposal of practitioners a new and very efficacious means of combating one of the gravest accidents of a preliminary to surgical operations, for which they cannot be too completely prepared.

Since chemical matches have come into general use, poisoning by arsenic, which formerly occurred so frequently, seldom comes under our notice. But the fatal facility of procuring phosphorus has, unfortunately, enabled guilty persons to replace it with advantage to their purpose. Criminal statistics prove that this substance is that most frequently employed in poisoning. Up to the present, medical science possessed no antidote to this poison, and its victims were fatally devoted to death.

M. Personne, a distinguished chemist, who has been long investigating this subject, has at length discovered an antidote to the disorganizing agent. Knowing, already, that essence of turpentine and other carburets of hydrogen deprive phosphorus of its property of becoming luminous in the darkness, and of burning at a low temperature, knowing that in the match manufactories at Stafford, in England, the workmen are protected from the necrosis resulting from the vapor of phosphorus by carrying a small phial of essence of turpentine attached to their breasts, M. Personne undertook a series of experiments which we detail for the benefit of your readers.

The experiments were fifteen in number, and were made in parallel series of three, on dogs of a middling size, and chosen, as much as possible, of the same strength. No. 1 of each series was given phosphorus alone. No. 2 was given the essence two hours after the poison. No. 3 received the antidote immediately after the injection of the phosphorus.

The essence and phosphorus were, in each case, conveyed to the stomach of the animal by means of an æsophageal catheter—the phosphorus always in a state of solution in a fatty substance. In one case it was administered in the shape of the paste used for making matches. The dose varied from one to three decigrammes.

No. 1 of each series, to which phosphorus alone had been given, making, in the five series, five animals, died.

Numbers 2, those animals to whom the antidote had been administered two hours after the injection of the poison, at first exhibited the same symptoms as the animals which had been given the poison alone, but finally, after copious discharges from the stomach, recovered, with one exception.

Numbers 3, finally, to which the antidote had been administered immediately after the poison, were only slightly indisposed, with the exception of one, which succumbed.

The animals belonging to series 2 and 3, which died, had absorbed 30 centigrammes of phosphorus, an enormous dose. In addition they had been placed in a temperature so cold as to freeze the water put at their disposal. Finally, notwithstanding the large quantity of phosphorus injected the antidote was administered in the same amount as in the other cases. Phosphorus kills by depriving the blood of the necessary oxygen, thus preventing its hæmatosis.

Essence of turpentine appears to possess the property, when absorbed, of preventing phosphorus from burning in the blood, as it prevents its combustion at a low temperatuae. The results of these experiments we regard as highly satisfactory, inasmuch as they give us a means of paralyzing the action of this poison, and finally eliminating it from the blood.

---

Some time ago M. Barbosa, of Lisbon, recommended sulphuret of potassium as a remedy for the croup. Dr. Labat and several other French physicians tried it without much success, notwithstanding the striking results which M. Barbosa had experienced in its employment. Dr. Labat had tried sulphuret of potassium as an expectorant, to combat the dry cough, which is so dangerous a stage in the disease. Not obtaining from this specific the results which he expected in the case of a little girl two years of age, Dr. Labat administered acetate of potash, which can be given in a large dose, and possesses the advantage of not having a strong flavor. He prescribed this salt in doses of 10

grammes dissolved in 120 grammes of water, to be taken by table spoonfuls every half hour. Three hours of this treatment produced an abundant expectoration, but, in this particular case, the secretion becoming purulent, the child succumbed. Convinced, however, that he had found a medicine capable of producing an abundant mucous expectoration, Dr. Labat administered acetate of potash with proportions of 8 grammes to 120 grammes of water to a child of 18 months, in which the cough had assumed an alarming dryness, twenty-four hours after tracheotomy. The following day, mucous expectorations had re-appeared, and the child recovered, after a treatment of 16 grammes of acetate of potash. Since this last case acetate of potash has been several times used under similar circumstances, notably, by Dr. Dudson, of St. Andrew's Hospital, at Bordeaux, and always with marked success.

Dr. Secorizat, physician to the Trans-Atlantic Navigation Company, has just published a remedy which he considers infallible for sea-sickness. It consists of faradization of the epigastric region, accompanied by the external use of a solution of atropine.

During the first day he does not attempt to arrest vomiting, when its frequency does not pass the limit natural to the temperament of the patient.

Experience has proved that if these evacuations are suddenly stopped at the commencement the result is an obstinate constipation. However, as soon as sea-sickness passes the limits of an ordinary indisposition, means of combating it should be at once sought. Especially in the case of persons of an extremely nervous temperament, as convulsions of a very grave nature might ensue.

When the illness assumes a serious character, Dr. Secorizat recommends friction of the epigastric region with a cloth dipped in water which may have a little soap dissolved in it; then, when this region is completely cleared of all greasy matter he uses the following lotion:

Sulphate of Atropine, 2 or 3 centigrammes.
Water, 30 grammes.

He afterwards applies a copper plate of four or five centimetres in diameter, in communication with one of the poles of a Rumkorff's Medical Aid within five or six centimetres of the navel in an upward and outward direction; the other meter, furnished with a wet sponge, is then moved from the hollow of the epigastrum to the plate, following the direction of the curves of the stomach. Five or six applications on each side generally suffice. These should be made as near as possible to the costal cartilage, without, however, touching them. The intensity of the current should be graduated according to the susceptibility of the patient, and the violence of the vomiting. It is sometimes advisable to use a small metallic pencil, instead of the plates, in order an energetic rubefaction and an efficacious revulsion. Since 1865, when Dr. Secorizat commenced these experiments he has had an opportunity of repeating them in several hundred cases, on persons of both sexes and of every social position. His success has been nearly always prompt and decisive.

Perhaps the means employed by Dr. Secorizat may be equally well adapted for checking vomiting peculiar to pregnancy, but in this case the physician should give the utmost attention to the effect of electricity on the general condition of the mother and child.

